# A Small “Tent” in the Esophagus

**DOI:** 10.5152/tjg.2024.23313

**Published:** 2024-07-01

**Authors:** Zeng-Min Zong, Jia-Yun Xu, Hai-Tao Zhang, Huan Xu, Xiao-Wei Tang, Lei Shi

**Affiliations:** 1 Department of Gastroenterology, The Affiliated Hospital of Southwest Medical University, Luzhou, China; 2 Department of Endoscopy Medicine, The Affiliated Hospital of Southwest Medical University, Luzhou, China

## CASE PRESENTATION

**Figure 1.**
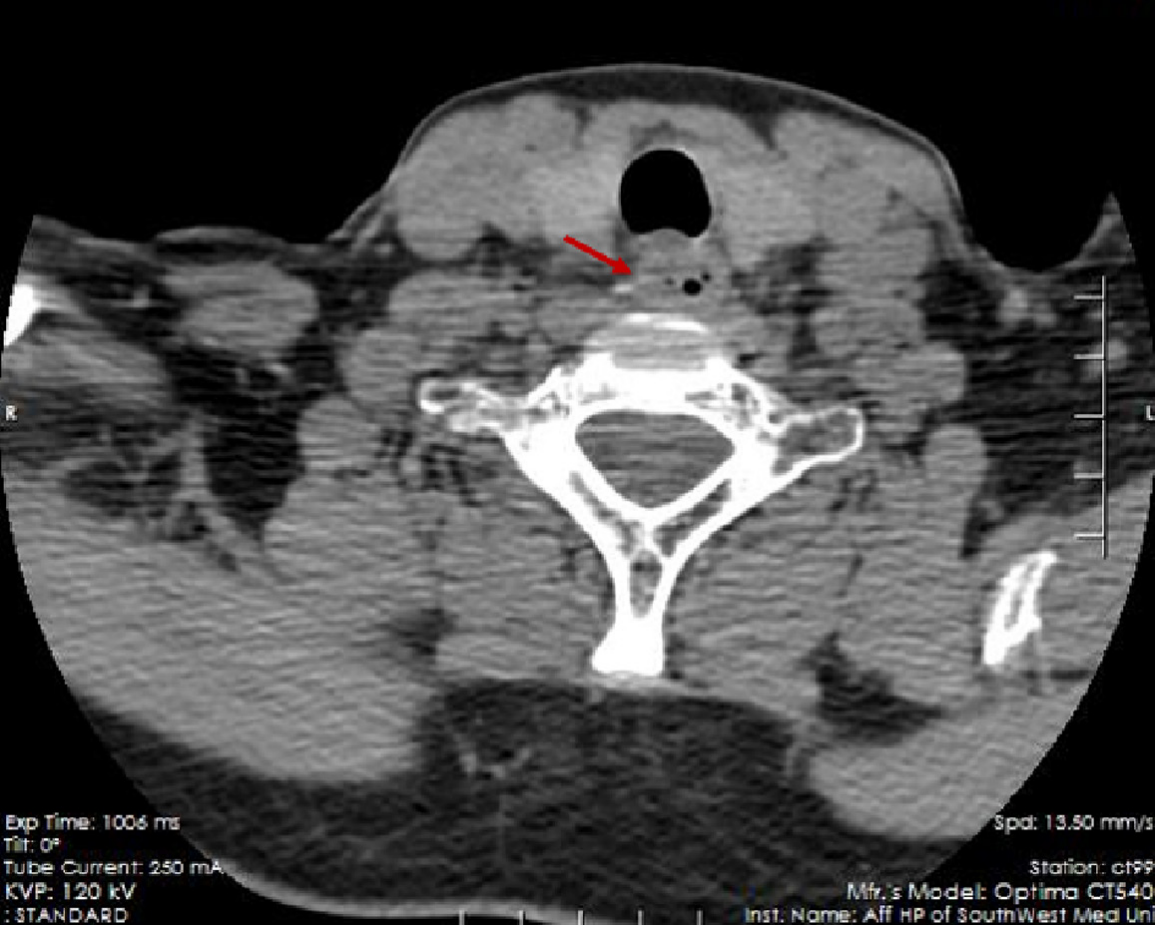
Computed tomography showed a dense linear shadow penetrating the anterior wall of the proximal esophagus (red arrow).

A 67-year-old woman was admitted to our hospital with a sore throat and retrosternal discomfort. Computed tomography (CT) showed a linear hyperdensity shadow penetrating the anterior wall of the proximal esophagus (Figure 1). During a comprehensive interview about her dietary history, the patient reported accidentally swallowing a fish bone 2 weeks ago. Next, we performed an esophagogastroduodenoscopy (EGD) that revealed a small “tent” with smooth surface mucosa in the upper esophagus, approximately 20 cm from the incisor teeth (Figure 1). Based on these examinations, a diagnosis of fishbone invasion into the esophageal submucosa was considered. 

## TECHNIQUE

**Figure 2.**
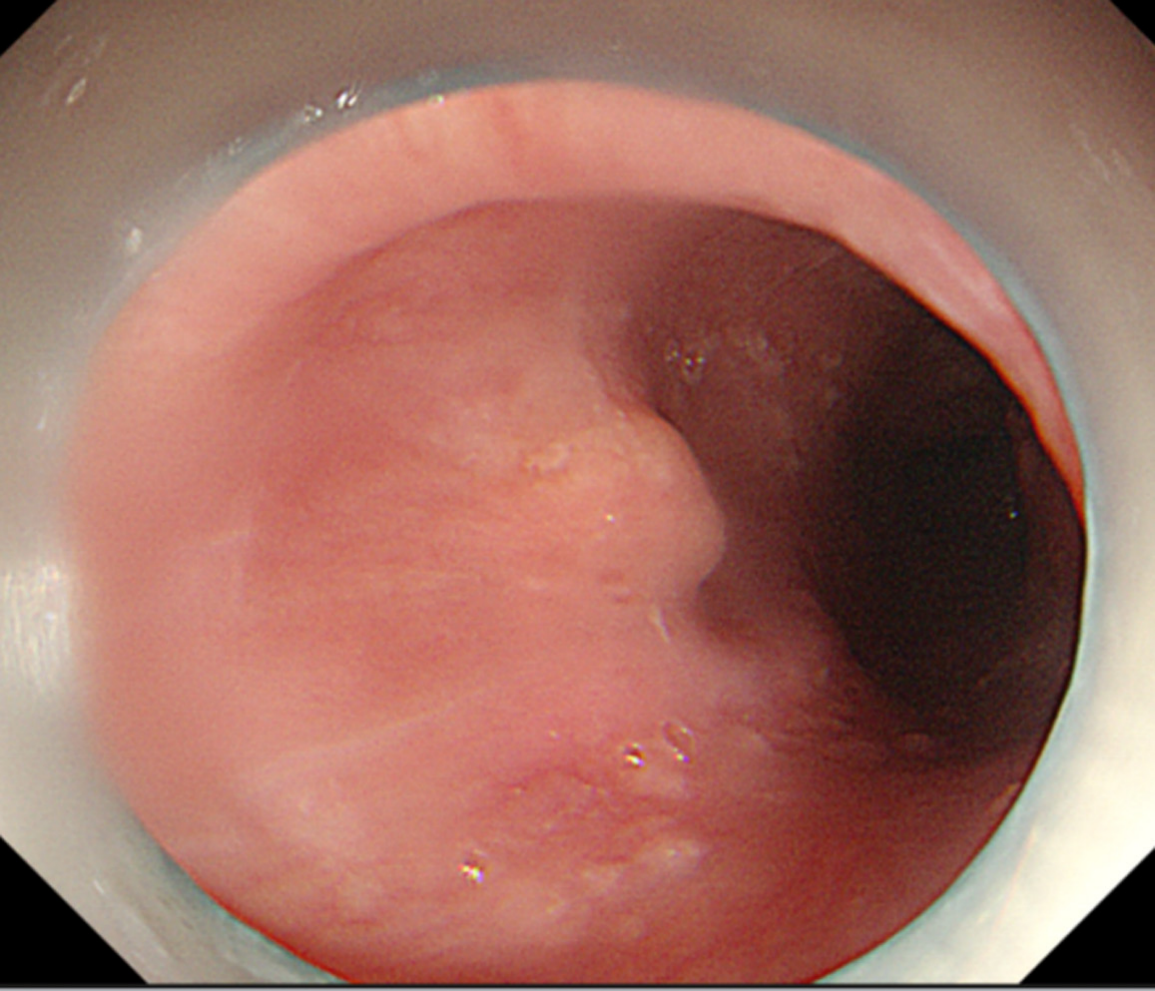
Esophagogastroduodenoscopy revealed a small “tent” in the upper esophagus with smooth surface mucosa.

**Figure 3.**
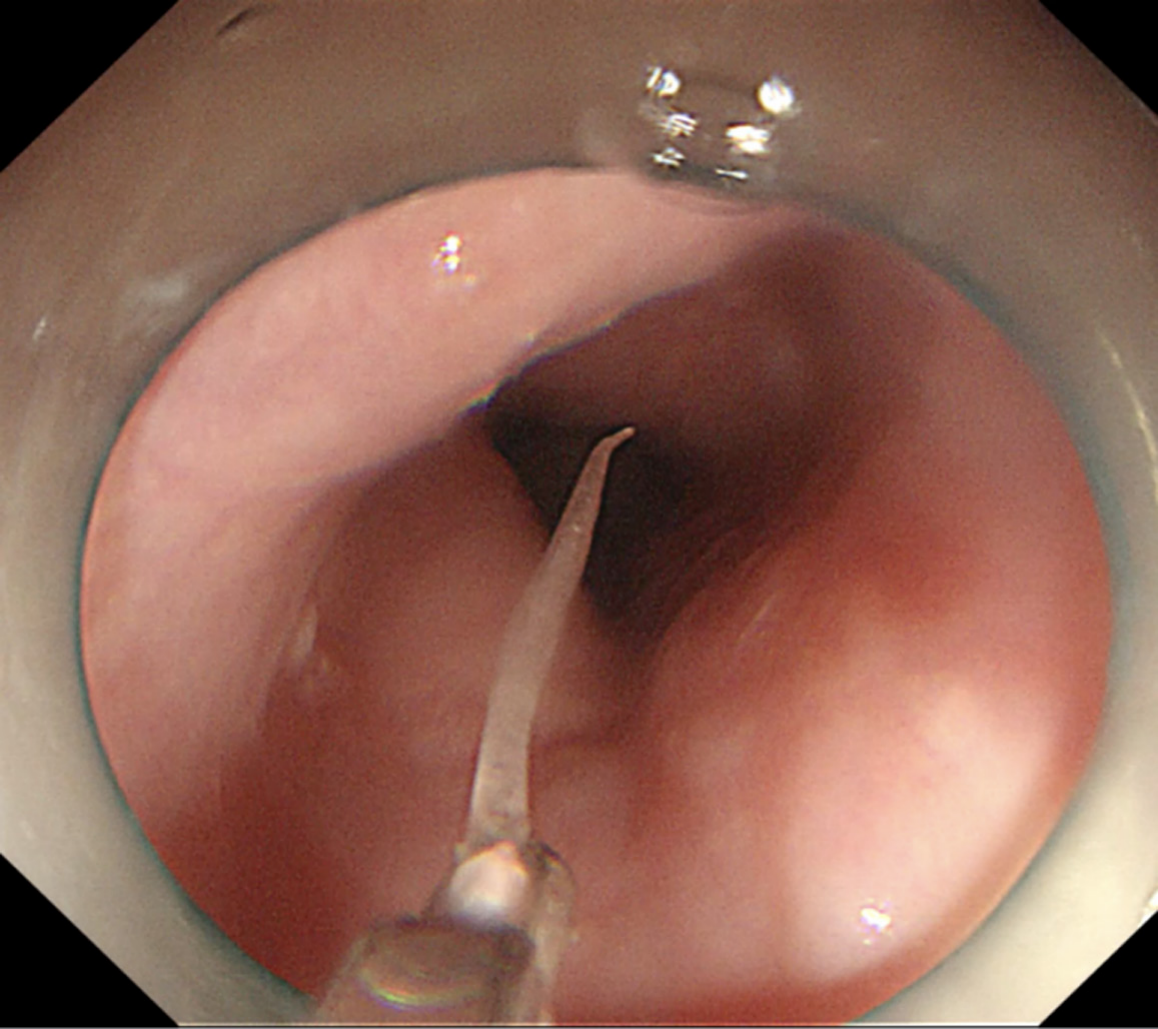
Extraction of the fish bone by monopolar hemostatic forceps.

**Figure 4.**
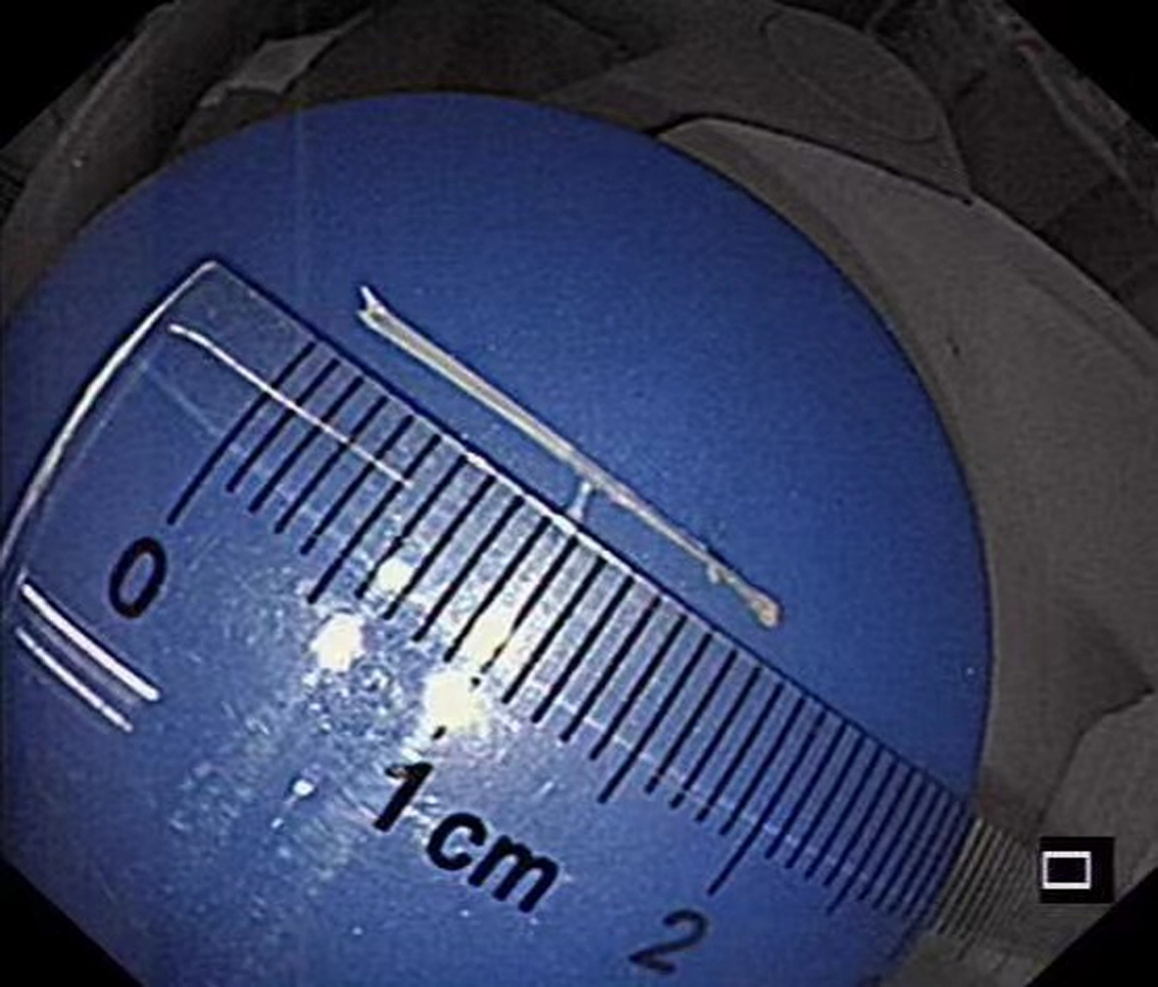
A 1.7 cm-long fish bone.

Initially, monopolar hemostatic forceps (FD-410LR; Olympus, Tokyo, Japan) were used to seize the mucosa of the “tent” and completed the mucosal incision. However, attempts to remove the fishbone with foreign body forceps were unsuccessful. Consequently, we switched to monopolar hemostatic forceps again, followed by dissecting the submucosa using the head of monopolar hemostatic forceps (endo cut Q mode, effect 3, time 2, and interval 4). This exposed the tip of the foreign body. Finally, a 1.7 cm-long fish bone was safely removed using the hemostatic forceps (Figures 2- 4). A clip was used to close the esophageal mucosal injury. The postoperative course was uneventful.

## CONCLUSION

Foreign body migration into the deeper tissue of the esophagus is a rare problem with a high risk of perforation.^1^ The management of entirely embedded foreign bodies remains challenging.^2^ Some studies suggest that endoscopic submucosal dissection (ESD) can be used to expose and remove foreign bodies under the guidance of endoscopic ultrasound (EUS).^3^ However, our case is a valuable example of an entirely submucosal fish bone case that could be treated by monopolar hemostatic forceps rather than ESD, which lessened medical expense and made our treatment plan as safe and effective as possible.

In conclusion, dietary history inquiries and the use of CT combined with EGD aid in making a diagnosis. For similar patients, monopolar hemostatic forceps may be preferred, allowing the patient to avoid the ESD procedure.

*The video file linked to this article is available in the online version of the journal. Or you can utilize the QR code provided on this page to gain access.
